# LOCAL PERIARTICULAR ANALGESIA IN TOTAL KNEE ARTHROPLASTY

**DOI:** 10.1590/1413-785220172502151116

**Published:** 2017

**Authors:** David Sadigursky, Daniel Pereira Simões, Raphael Araújo de Albuquerque, Monize Zórnio Silva, Rogério Jamil Carneiro Fernandes, Paulo Oliveira Colavolpe

**Affiliations:** 1 Clínica Ortopédica e Traumatológica (COT), Salvador, BA, Brasil.; 2 Faculdade de Ciências e Tecnologia (FTC), Salvador, BA, Brasil.

**Keywords:** Analgesia, Arthroplasty, Knee, Seepage

## Abstract

**Objective:**

To evaluate the use of infiltration of periarticular analgesic agents intraoperatively in total knee arthroplasty (TKA), with regard to benefits, reduction of pain, opioid consumption, improvement of range of motion and early ambulation.

**Methods:**

To analyze the benefits of periarticular drug infiltration, the patients submitted to TKA were evaluated, being separated into two groups. One group received the local periarticular infiltration protocol containing 0.5% bupivacaine (400mg/20ml), 1/1000 epinephrine (0.3ml), triamcinolone hexacetonide (20mg/1ml), clonidine (150mcg/1ml) and 20 ml of saline (0.9% SS) and, the other group underwent conventional intravenous analgesia. The results were compared and the variables analyzed were age, sex, BMI, comorbidities, postoperative complications, pain, functional capacity, range of motion, transfusion and rescue opioids for analgesia.

**Results:**

The mean age of the patients was 68 years and most were female and presented involvement of the left knee. Postoperatively, patients who had received periarticular infiltration showed improvement of pain as well as functional capacity.

**Conclusion:**

The analysis of data obtained demonstrated that the periarticular infiltration of analgesic agents is significantly effective for pain control and functional recovery.***Level of Evidence II, Prospective Comparative Study.***

## RESUMO

**Objetivo:**

Avaliar a realização da infiltração de solução de agentes analgésicos periarticulares no intraoperatório da artroplastia total do joelho (ATJ), no que tange aos seus benefícios, redução da dor, consumo de opioides, melhora do arco de movimento e deambulação precoce.

**Métodos:**

Para avaliar os benefícios da infiltração de agentes periarticulares, foram analisados pacientes submetidos à ATJ, sendo separados em dois grupos. Um grupo recebeu o protocolo de infiltração periarticular local, contendo solução de bupivacaína a 0,5% (400 mg /20 ml), epinefrina 1/1000 (0,3 ml), hexacetonido de triancinolona (20 mg/1 ml), clonidina (150 mcg/1 ml) e 20 ml de solução salina (SF a 0,9%) e outro grupo recebeu analgesia endovenosa convencional. Os resultados foram comparados e as variáveis analisadas foram idade, sexo, IMC, comorbidades, complicações pós-cirúrgicas, dor, capacidade funcional, amplitude de movimento, transfusão e resgate de opioides para analgesia.

**Resultados:**

Dos pacientes analisados a média de idade foi de 68 anos e a maioria era do sexo feminino e com acometimento do joelho esquerdo. No pós-operatório os pacientes que haviam recebido infiltração periarticular apresentaram melhora da dor, bem como da capacidade funcional.

**Conclusão:**

A análise dos dados obtidos demonstrou que a infiltração periarticular de agentes analgésicos é significativamente eficaz para o controle da dor e recuperação funcional. Nível de Evidência II, Estudo Prospectivo Comparativo.

## INTRODUCTION

Many surgical procedures performed in orthopedics involve an extremely complex pain mechanism, and a number of studies have demonstrated that further study is needed on the topic of controlling perioperative pain to achieve more effective pain control.^1^ ^-^ ^4^

We know that surgical patients who receive appropriate analgesia are more adherent to postoperative rehabilitation programs. One of the main goals of postoperative analgesia is improvement in functional results and early return to routine activities, improving patient quality of life.^5^

The growing number of total knee arthroplasties and increased life expectancy reinforce the need for early rehabilitation to completely restore function in the operated joint and improve pain with the fewest complications.^6^ ^,^ ^7^ In order to solve this problem, a number of studies are carried out each year to find a viable solution that will improve postoperative pain in patients.^3^ ^,^ ^4^ ^,^ ^8^

Our objective was to assess postoperative improvement after total knee arthroplasty by comparing the use of multimodal periarticular infiltration with analgesic agents with conventional analgesia. More specifically, the objective of this study was to compare the postoperative analgesia obtained through periarticular infiltration of a solution containing 0.5% bupivacaine (400mg/20ml), 1/1000 epinephrine (0.3ml), triamcinolone hexacetonide (20mg/1ml), clonidine (150mcg/1ml), and 20ml of 0.9% saline solution with the use of intravenous analgesia utilizing opiates and painkillers (tramadol 100mg and dipyrone 1g) according to the assessment of opioid analgesic consumption, function (using the WOMAC scale), and evaluation of pain (using the VAS scale).

## METHODS

To evaluate the results, we conducted a prospective and comparative study from March 2008 to December 2014. We selected 59 patients with a diagnosis of primary osteoarthritis of the knee who underwent elective surgeries for TKA, and separated them into two groups using permuted block randomization.^9^

The study began after approval by the institutional review board, and was registered under process number CAAE 38426214.5.0000.5032. All patients signed an informed consent form before being included in the study.

Participants were male and female patients aged 60 to 80 years, with grade 3 or above according to the Kellgren and Lawrence classification,^10^ indicated for TKA with no bone defects requiring additional grafts or implants, and did not have pronounced angular deformities.

We excluded patients with psychiatric disorders, dependence on alcohol or illegal drugs, allergies to morphine, dipyrone or any local anesthetic, previous infection in the knee or other joints, systemic inflammatory diseases, congenital deformities or neurological disorders, and arthroplasty revision.

The variables evaluated were: age, sex, body mass index (BMI), post-surgical complications, pain level evaluated by the analog pain scale (VAS), ability to ambulate, time to begin walking, range of motion, and the need for transfusion and oral and intravenous rescue opioid analgesia.

The patients who underwent TKA were divided into two groups: Group 1 (29 individuals) received spinal anesthesia as well as the trans-operative analgesia protocol using infiltration of multimodal periarticular analgesic drugs. Group 2 (30 subjects) received only conventional intravenous analgesia using opiates and painkillers (tramadol 100mg and dipyrone 1g) and morphine sulfate (4mg every 2 hours) as required by the patient, which was similar in both groups. The drugs used in the spinal anesthesia were the same for all patients according to the anesthesia team protocol, namely 0.5% isobaric bupivacaine (5mg) and morphine hydrochloride (0.1mg), and were administered by the same anesthesia team. All patients who required medications outside the anesthesia team protocol for TKA were excluded from the study.

All patients received the same pre-emptive analgesia containing dipyrone (1g every 6 hours), tramadol (100mg every 8 hours), and pregabalin (75mg every 12 hours). The patients were oriented on the use of medications 24 hours prior to the surgery, and the drugs were maintained during hospitalization.

The medications used for intraoperative analgesia by the individuals in Group 1 are listed in Table 1.

**Table 1:** Multimodal drugs used for periarticular knee infiltration.

Bupivacaine 0.5%	400 mg/20ml
Epinephrine 1/1000	1/1000 (0.3ml) 300 Mcg
Triamcinolone hexacetonide	20mg/1ml
Clonidine	150mcg/1ml
Saline solution (0.9%)	20ml

The drugs used for periarticular infiltration were based on the studies by Ranawat.^11^

The medications used in the postoperative period for both groups were: dipyrone (1g IV every 6 hours), tramadol (100mg orally every 8 hours) as needed, and morphine sulfate (4mg every 2 hours) as requested for persisting pain above 7 on the visual analog scale (VAS). Extra rescue doses of opioids were recorded (usage and frequency).

Pain intensity was evaluated in three periods: prior to surgery, 24 hours after the procedure, and 48 hours after the procedure.

A pneumatic tourniquet at 100 mmHg above systolic blood pressure was used to control bleeding during the surgical procedure in all cases. A suction drain was used for 24 hours. After cementing and placement of the implants, periarticular infiltration was conducted using the drugs listed in Table 1.

Before placement of the polyethylene component, periarticular infiltration was performed in the following order: posterior capsule, posterior-lateral and posterior-medial structures, patellar ligament and quadriceps tendon, synovia, capsule, pes anserinus, periosteum, iliotibial band, and tibial and fibular collateral ligaments at their origins. Rehabilitation began on the first day after surgery and the protocol was identical in both groups. Assessment began 6 hours after the end of the procedure.

The quantitative variables were presented as means and their standard deviations, medians, and interquartile ranges. Categorical variables were represented using frequencies and percentages. Numerical variables were compared between groups using Student's T test for variables that assumed normal distribution and the Mann-Whitney U test for variables with non-normal distribution. Proportions were compared using the chi-squared test or Fisher's test (when necessary), and ANOVA was conducted on repeated measurements considering the VAS score at three points in time (pre-procedure, 24 hours post-procedure, and 48 hours post-procedure) between the groups (with or without periarticular infiltration), analyzing the clinically significant variables and/or those which exhibited significant differences or trends in univariate analysis: preoperative WOMAC and opioid rescue dose at 48 hours. The analyses were conducted using IBM Statistical Package for the Social Sciences software version 20.0 (SPSS, Chicago, IL, USA) and the R programming language and environment

(R Core Team, 2014).

## RESULTS

A total of 59 patients were selected, 36 (61%) females and 23 (39%) males. Mean patient age was 68, (65.0-75.0) and mean patient BMI was 27.0 (65.0-75.0).

Table 2 presents the clinical data for the patients studied. Of the total, 30 patients (50.8%) were affected in the right knee and 29 (49%) in the left knee. Identified comorbidities are shown in Table 2.

**Table 2:** Clinical data for patients undergoing TKA from 2008 to 2014.

Characteristic	Total (n = 59)	Without infiltration (n = 30)	Periarticular injection (n = 29)
Side affected			
Right	30.0 (50.8)	16.0 (53.3)	14.0 (48.3)
Left	29.0 (49.0)	14.0 (46.7)	15.0 (51.7)
Comorbidities			
High blood pressure (HBP)	30.0 (50.8)	13.0 (43.3)	17.0 (58.6)
Diabetes mellitus	10.0 (16.9)	3.0 (10.0)	7.0 (24.1)
Dyslipidemia	5.0 (8.5)	2.0 (6.7)	3.0 (10.3)
Controlled Chronic Kidney Disease	2.0 (3.4)	1.0 (3.3)	1.0 (3.4)
Previous Stroke	2.0 (3.4)	2.0 (6.7)	0.0 (0.0)
Gastroesophageal Reflux Disease	1.0 (1.7)	1.0 (1.6)	0.0 (0.0)

All data are presented as n (%).

Table 3 presents the clinical data related to the surgery. Lower scores on the Visual Analog Scale for pain were seen in patients in Group A (3.7-3.9) 24h post-procedure as well as 48h post-procedure, respectively; group B presented higher values on the pain scale (5.3 at 24h post-procedure and 4.8 at 48h post-procedure). The partial load (with aids) was evaluated by examining the standing patient supported by a walker while exercising with the assistance of the physical therapist. In this test, higher mean scores were seen at 24 and 48h (10.3 and 11.0%) in the patients in group A and at 24h and 48 hours post-procedure (10 and 12%) in the patients in group B. The WOMAC assessment of functional capacity showed no statistically significant difference between the two groups in the postoperative period of 3 month. Clinical complications are presented in Table 3.

**Table 3:** Data relating to surgery for groups of patients undergoing TKA from 2008 to 2014.

Visual analog Pain scale	Total (n = 59)	Without infiltration (n = 30)	Periarticular injection (n = 29)	p value
(Mean ± Standard deviation)				
Preoperative	7.9 ± 0.8	7.7 ± 0.6	8.1 ± 0.9	0.065
24h Postoperative	4.5 ± 1.2	5.3 ± 0.9	3.7 ± 0.9	< 0.001
48h Postoperative	4.3 ± 1.2	4.8 ± 1.1	3.9 ± 1.2	0.004
Functional Capacity				
WOMAC (Mean ± Standard deviation)				
Preoperative	28.6 ± 7.1	26.0 ± 5.2	31.2 ± 7.9	0.004
Postoperative (3 months)	47.3 ± 7.0	47.1 ± 6.6	47.4 ± 7.5	0.865
Rescue opioid				
24h	150.0 (100.0 - 300.0)	200 (100.0 - 300.0)	150.0 (100.0 - 200.0)	0.123
48h	100 (50.0 - 150.0)	150 (100.0 - 200.0)	50 (0.0 - 100.0)	< 0.001
Complications				
Nausea	9.0 (15.3)	6.0 (20.0)	3.0 (10.3)	0.472
Vomiting	5.0 (8.5)	4.0 (13.3)	1.0 (3.4)	0.353
Headache	4.0 (6.8)	4.0 (13.3)	0.0 (0.0)	0.112
Incontinence	2.0 (3.4)	0.0 (0.0)	2.0 (6.9)	0.237
Range of motion				
Preoperative (medians, extension - flexion)	0 - 120	0 - 120	0 - 120	
Postoperative (medians, extension - flexion)	0 - 100	0 - 100	0 - 110	

a - Student's t test; b - Chi-squared test; c - Mann-Whitney U test; d - Fisher's exact test.

Table 4 shows the VAS pain values in the group that did not receive infiltration, measured prior to the procedure, at 24h post-procedure, and at 48h post-procedure. These values were considerably higher, especially after the surgery, when compared to the group that received periarticular injection of the painkillers. After the surgery, at 24h and 48h post-procedure the values were significantly lower in the periarticular injection group (3.7 ± 0.2; 3.7 ± 0.2).

**Table 4:** Estimated means according to the ANCOVA model.

Visual analog pain scale	Without infiltration (n = 30)	Periarticular injection (n = 29)
(Mean ± Standard deviation)		
Preoperative	7,8 ± 0,1 (7,5 - 8,1)	8,1 ± 0,2 (7,7 - 8,4)
24h Postoperative	5,3 ± 0,2 (4,9 - 5,7)	3,7 ± 0,2 (3,3 - 4,1)
48h Postoperative	4,8 ± 0,2 (4,3 - 5,3)	3,7 ± 0,2 (3,3 - 4,3)

Notes: a - Controlling for preoperative WOMAC and rescue opioid in 48 hours; All values ​​are presented as mean ± standard deviation (95% confidence interval). MPA = Multimodal Periarticular Analgesia.

**Figure 1:**
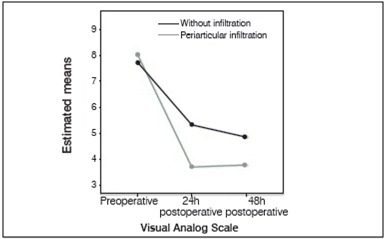
Mean values for VAS pre and postoperative estimated from ANCOVA of repeated measurements from patients who underwent TKA at the Clínica Ortopédica e Traumatológica in Salvador, Bahia from 2008 to 2014.

Figure 1 contains data relating to analysis of the Visual Analog Scale for pain over time and between the groups. In both groups, a continuous decrease was seen in the postoperative periods at 24h and 48h, and was more pronounced in the group that underwent the procedure. The VAS scale demonstrates statistically significant values at the different points in time which were analyzed. 

## DISCUSSION

The results of this prospective randomized clinical study showed significant pain reduction as assessed by the VAS with statistically significant data, especially in the first 24 hours post-procedure, showing the benefit of periarticular anesthetic infiltration in pain reduction in the immediate postoperative period up to 48 hours post-procedure. Pain relief and functional recovery after surgery using the protocol described presented excellent security for pain control and functional recovery.

Superior pain relief provided by infiltration of analgesics for pain control over exclusively intravenous analgesia was demonstrated in a systematic review conducted by Jiang et al.^6^ Twenty-one studies were included in this analysis; lack of standardization in the protocols for periarticular injection with multimodal drugs with regard to dose and application site was considered the most significant limitation of this meta-analysis.

Vendittoli et al.^12^ demonstrated that the use of periarticular infiltration with multimodal drugs could result in less pain, improved functional recovery, and patient satisfaction. However, Koh et al.^13^ observed that pain reduction was significant in the immediate postoperative period, with no improvement in functional results or patient satisfaction after 48 hours.

Currently, multimodal management of perioperative pain has been the most common means of reducing the incidence of persistent postoperative pain. It should be noted that intra-articular analgesia has a fleeting effect and is not a substitute for other therapies after hospital discharge.^1^ ^,^ ^3^ ^,^ ^13^ ^,^ ^14^.

Nevertheless, intra-articular intraoperative injection of multimodal analgesics has shown significant results in enhancing the analgesic effect without increased complications resulting from the use of oral or intravenous opioids. This control may significantly reduce the need to use opioids, and may improve patient satisfaction without apparent risks in the period following TKA surgery.^15^ The greatest benefit demonstrated in this study as well as others found in the literature is reduced consumption of opioid medications such as tramadol and morphine sulfate. These drugs are frequently required in patients undergoing TKA because of the pain experienced by a large proportion of patients during this period.

Pharmaceutical synergy produces more effective analgesia by addressing pain through all its mechanisms. Epinephrine prolongs the action of local agents by decreasing absorption via vasoconstriction through its α-adrenergic effects. It can also reduce bleeding and postoperative hematoma. Morphine has central, regional, and local effects via its effect on opioid receptors. Local administration yields reduces the frequency of typical opioid side effects (for example, sedation, nausea, and respiratory depression) which occur through the opioid receptors. Clonidine works through its α2-adrenergic actions. This strengthens the action of local anesthetics and opioids through synergistic effects. By suppressing these physiological responses to surgery, pain and functional recovery are improved.^15^ ^-^ ^18^ ^).^

Limitations of this study include the sample in comparison with multi-center studies, and the focus on a specific and homogenous population. Further studies should be conducted in order to compare the analgesic drugs, defining the best protocol.

## CONCLUSION

Use of periarticular infiltration in total knee arthroplasty reduces pain and improves functional capacity in the 48-hour period immediately following surgery when compared to oral and intravenous analgesia alone. We did not observe an increase in the incidence of side effects when the multimodal drug protocol was used on periarticular infiltration.

## References

[B1] Webb CA, Mariano ER (2015). Best multimodal analgesic protocol for total knee arthroplasty. Pain Manag.

[B2] Tietje T, Davis AB, Rivey MP (2015). Comparison of 2 Methods of Local Anesthetic-Based Injection as Part of a Multimodal Approach to Pain Manag ement After Total Knee Arthroplasty. J Pharm Pract.

[B3] Parvataneni HK, Shah VP, Howard H, Cole N, Ranawat AS, Ranawat CS (2007). Controlling Pain After Total Hip and Knee Arthroplasty Using a Multimodal Protocol With Local Periarticular Injections. J Arthroplasty.

[B4] Nakai T, Tamaki M, Nakamura T, Nakai T, Onishi A, Hashimoto K (2013). Controlling pain after total knee arthroplasty using a multimodal protocol with local periarticular injections. J Orthop.

[B5] Tanaka N, Sakahashi H, Sato E, Hirose K, Ishii S (2001). The efficacy of intra-articular analgesia after total knee arthroplasty in patients with rheumatoid arthritis and in patients with osteoarthritis. J Arthroplasty.

[B6] Jiang J, Teng Y, Fan Z, Khan MS, Cui Z, Xia Y (2013). The efficacy of periarticular multimodal drug injection for postoperative pain management in total knee or hip arthroplasty. J Arthroplasty.

[B7] Springer BD (2014). Transition from nerve blocks to periarticular injections and emerging techniques in total joint arthroplasty. Am J Orthop Belle Mead NJ.

[B8] Jiang J, Teng Y, Fan Z, Khan MS, Cui Z, Xia Y (2013). The Efficacy of Periarticular Multimodal Drug Injection for Postoperative Pain Manag ement in Total Knee or Hip Arthroplasty. J Arthroplasty.

[B9] Gail MH, Mark SD, Carroll RJ, Green SB, Pee D (1996). On design considerations and randomization-based inference for community intervention trials. Stat Med.

[B10] Kellgren JH, Lawrence JS (1957). Radiological Assessment of Osteo-Arthrosis. Ann Rheum Dis.

[B11] Ranawat AS, Ranawat CS (2007). Pain management and accelerated rehabilitation for total hip and total knee arthroplasty. J Arthroplasty.

[B12] Vendittoli P-A, Makinen P, Drolet P, Lavigne M, Fallaha M, Guertin M-C (2006). A multimodal analgesia protocol for total knee arthroplasty. A randomized, controlled study. J Bone Joint Surg Am.

[B13] Koh IJ, Kang YG, Chang CB, Kwon SK, Seo ES, Seong SC (2010). Additional pain relieving effect of intraoperative periarticular injections after simultaneous bilateral TKA: a randomized, controlled study. Knee Surg Sports Traumatol Arthrosc Off J ESSKA.

[B14] Busch CA, Shore BJ, Bhandari R, Ganapathy S, MacDonald SJ, Bourne RB (2006). Efficacy of periarticular multimodal drug injection in total knee arthroplasty. A randomized trial. J Bone Joint Surg Am.

[B15] Wang C, Cai X-Z, Yan S-G (2015). Comparison of Periarticular Multimodal Drug Injection and Femoral Nerve Block for Postoperative Pain Manag ement in Total Knee Arthroplasty : A Systematic Review and Meta-Analysis. J Arthroplasty.

[B16] Zhao X, Qin J, Tan Y, Mohanan R, Hu D, Chen L (2015). Efficacy of steroid addition to multimodal cocktail periarticular injection in total knee arthroplasty: a meta-analysis. J Orthop Surg.

[B17] Milani P, Castelli P, Sola M, Invernizzi M, Massazza G, Cisari C (2015). Multimodal Analgesia in Total Knee Arthroplasty : A Randomized, Double-Blind, Controlled Trial on Additional Efficacy of Periarticular Anesthesia. J Arthroplasty.

[B18] Elmallah RK, Cherian JJ, Pierce TP, Jauregui JJ, Harwin SF, Mont MA (2016). New and Common Perioperative Pain Manag ement Techniques in Total Knee Arthroplasty. J Knee Surg.

